# Clinical Utility of Breast Ultrasound Images Synthesized by a Generative Adversarial Network

**DOI:** 10.3390/medicina60010014

**Published:** 2023-12-21

**Authors:** Shu Zama, Tomoyuki Fujioka, Emi Yamaga, Kazunori Kubota, Mio Mori, Leona Katsuta, Yuka Yashima, Arisa Sato, Miho Kawauchi, Subaru Higuchi, Masaaki Kawanishi, Toshiyuki Ishiba, Goshi Oda, Tsuyoshi Nakagawa, Ukihide Tateishi

**Affiliations:** 1 Department of Diagnostic Radiology, Tokyo Medical and Dental University Hospital, 1-5-45, Yushima, Bunkyo-ku, Tokyo 113-8501, Japan; 2 Department of Radiology, Dokkyo Medical University Saitama Medical Center, 2-1-50 Minami-koshigaya, Koshigaya 343-8555, Japan; 3 Department of Surgery, Breast Surgery, Tokyo Medical and Dental University Hospital, 1-5-45, Yushima, Bunkyo-ku, Tokyo 113-8501, Japan

**Keywords:** breast cancer, ultrasound, artificial intelligence, deep learning, generative adversarial networks, generative artificial intelligence

## Abstract

Background and Objectives: This study compares the clinical properties of original breast ultrasound images and those synthesized by a generative adversarial network (GAN) to assess the clinical usefulness of GAN-synthesized images. Materials and Methods: We retrospectively collected approximately 200 breast ultrasound images for each of five representative histological tissue types (cyst, fibroadenoma, scirrhous, solid, and tubule-forming invasive ductal carcinomas) as training images. A deep convolutional GAN (DCGAN) image-generation model synthesized images of the five histological types. Two diagnostic radiologists (reader 1 with 13 years of experience and reader 2 with 7 years of experience) were given a reading test consisting of 50 synthesized and 50 original images (≥1-month interval between sets) to assign the perceived histological tissue type. The percentages of correct diagnoses were calculated, and the reader agreement was assessed using the kappa coefficient. Results: The synthetic and original images were indistinguishable. The correct diagnostic rates from the synthetic images for readers 1 and 2 were 86.0% and 78.0% and from the original images were 88.0% and 78.0%, respectively. The kappa values were 0.625 and 0.650 for the synthetic and original images, respectively. The diagnoses made from the DCGAN synthetic images and original images were similar. Conclusion: The DCGAN-synthesized images closely resemble the original ultrasound images in clinical characteristics, suggesting their potential utility in clinical education and training, particularly for enhancing diagnostic skills in breast ultrasound imaging.

## 1. Introduction

Breast cancer remains a significant global health challenge and is the most commonly diagnosed cancer in women worldwide. Historically, the global incidence of breast cancer has been rising, partly due to increased life expectancy and lifestyle changes. In the early 20th century, breast cancer was relatively less common, but by the late 20th and early 21st centuries, it emerged as one of the most frequently diagnosed cancers. This trend is particularly evident in developed countries, where widespread screening and awareness campaigns have contributed to earlier detection. In 2020 alone, approximately 2.26 million cases were recorded globally, and about 685,000 women worldwide died from breast cancer, making it the leading cause of cancer death among women [1]. However, despite the increase in incidence, mortality rates have gradually declined in many developed countries since the 1990s, largely due to improved treatment modalities and effective screening programs. The 5-year survival rate for breast cancer in these countries is now over 80%, underscoring the importance of early detection and diagnosis in providing effective treatment [1].

One breast cancer screening modality is ultrasonography, which we widely use because it is minimally invasive and low-cost [2]. Meanwhile, diagnostic accuracy varies because it depends on experience and individual competence. Although beginners need to learn by assessing many images, medical images may contain personal information, which limits experience with cases outside of the institution and opportunities to encounter images of infrequent diseases.

Artificial Intelligence (AI) in medical fields, particularly in breast ultrasound imaging, plays a critical role in enhancing diagnostic accuracy. Lesion detection involves AI algorithms identifying abnormal areas or tissues, thereby aiding in early disease recognition. Segmentation, another crucial function, refers to the process where AI delineates the contours of lesions, separating them from normal tissue for precise analysis. High diagnostic rates signify AI’s effectiveness in correctly diagnosing conditions based on imaging data, reducing errors and improving patient outcomes. AI’s integration into medical imaging thus represents a significant advancement in diagnostic techniques, offering more accurate, efficient, and reliable evaluations [3,4,5,6,7,8]. Additionally, AI has been found to be useful for diagnosing axillary lymph node metastasis and predicting lymph node metastasis in breast cancer [9,10]. Ozaki et al. reported on the use of a deep learning model, employing convolutional neural networks for differentiating between normal and metastatic axillary lymph nodes in breast ultrasound images. The model, trained on over 600 images, achieved a sensitivity of 94%, a specificity of 88%, and an area under the curve of 0.966. Its diagnostic performance was comparable to an experienced radiologist and superior to less experienced readers. With assistance, the diagnostic accuracy of the residents improved significantly, demonstrating the model’s potential as an effective diagnostic aid in breast ultrasound imaging [9]. Zhou et al. reported on the feasibility of using deep learning to predict axillary lymph node metastasis in primary breast cancer patients from ultrasound images. The study used images from two hospitals, training three convolutional neural networks on one dataset and testing on both. The best-performing model, Inception V3, achieved an AUC of 0.89, with 85% sensitivity and 73% specificity, outperforming radiologists in diagnosis. This suggests that deep learning models can effectively predict clinically negative axillary lymph node metastasis, offering a potential early diagnostic tool for breast cancer patients [10].

In recent years, significant progress has been made in AI techniques for medical image synthesis [11,12,13,14]. Among them, an image-generation model called the generative adversarial network (GAN) was published in 2014 and has attracted much attention [15]. GAN involves two networks that are trained simultaneously as neural network models, with one performing image-generation and the other performing discrimination of the generated image to produce a realistic virtual image [16]. In recent studies, GANs produced high-quality medical images, and they have been the subject of several active studies, including optic-nerve papillary optical coherence tomography image synthesis for glaucoma detection [17], 3D magnetic resonance angiography cerebro-vascular image synthesis [18], skin-lesion segmentation [19], and chest X-ray image synthesis for pneumonia diagnosis [20]. Since the announcement of Chat GPT (Chat Generative Pre-trained Transformer, developed by OpenAI, San Francisco, CA, USA) in November 2022, generative AI has rapidly gained popularity as a form of social media. Chat GPT is a state-of-the-art large-scale language model that evaluates user input to simulate human-like conversations [21]. Large-scale language models are revolutionizing various fields, including healthcare. In medicine, they assist in analyzing patient data, generating medical reports, and providing diagnostic suggestions. Their ability to understand and process complex medical literature and patient information can support doctors in decision-making. However, challenges like data sensitivity, the need for highly accurate and unbiased outputs, and ethical considerations are crucial. The integration of these models in healthcare promises to enhance patient care and medical research, but it requires careful implementation to address potential risks. The Microsoft Corporation (Washington, USA) and OpenAI are studying the use of ChatGPT for clinical support and medical education [22].

In previous studies, we have used a deep convolutional GAN (DCGAN) [23], an adversarial generative network using deep learning, to generate breast mass synthetic images. These are the first successfully generated breast ultrasound images so realistic that even radiologists could not distinguish between the synthetic and original images [24]. The second study demonstrated DCGAN’s capability to produce high-quality synthetic ultrasound images of normal, benign, and malignant breast tissues, highlighting its potential in creating realistic virtual representations of tumor development, including growth and malignant transformation processes [25]. However, in these previous studies, the synthetic breast ultrasound images were generated without separating the images by tissue type. Meanwhile, in the present study, the DCGAN was used to generate breast ultrasound synthetic images by pathological diagnosis of tissue type. The generated images were evaluated by a radiologist along with the actual images and compared in detail. The evaluation focused on whether the generated images looked realistic and adequately represented each tissue’s characteristics. Furthermore, the possibility of creating a generated image for each pathological tissue was discussed to see if it could be used to improve physicians’ diagnostic skills and for educational purposes. Two radiologists meticulously examined both the original and generated images, subsequently scrutinizing correct response rates pertaining to histological categorization and the benign or malignant assessment of tumor images. Further, an assessment of concordance between the original and the synthetic images was conducted utilizing the kappa coefficient.

This investigation underscores the efficacy of our DCGAN model in generating images closely resembling the originals. These generated images hold potential utility for medical students and fledgling physicians in honing their skills in interpreting breast ultrasound images.

## 2. Materials and Methods

### 2.1. Patients

The subjects were patients who underwent breast ultrasound examinations at Tokyo Medical and Dental University Hospital from September 2014 to August 2022. The inclusion criteria were female patients whose masses were diagnosed as benign or malignant by pathological analysis or by >1-year follow-up examinations at our hospital. The exclusion criteria were (a) patients who were treated with hormonal therapy, chemotherapy, or radiation therapy, (b) patients who were <20 years old, and (c) patients who were unable to express their consent due to illness or advanced age.

Our medical ethics committee approved this retrospective study and waived the requirement for written informed consent from patients (approval number: M2019-232; approval date: 13 December 2019). All methods were carried out in accordance with relevant guidelines and regulations (Declaration of Helsinki).

### 2.2. Ultrasound Examinations

Five radiologists with 4–20 years of experience performed the ultrasound examinations on an Aplio XG scanner with an 8.0 MHz linear probe PLT-805AT (Toshiba Medical Systems, Tochigi, Japan), an Aplio 500 scanner with an 8.0 MHz linear probe PLT-805AT (Toshiba Medical Systems, Tochigi, Japan), or a LOGIC E10s scanner with a linear matrix probe ML6-15-D (GE Healthcare, Chicago, IL, USA). The radiologists acquired static images in the vertical and horizontal planes and measured the maximum diameter of the masses. The investigator was aware of the clinical and mammographic findings at the time of the ultrasound examination. The patients underwent the examination in a supine position with their arms down.

### 2.3. Data Set

One medical student and one radiologist specializing in breast cancer imaging extracted typical breast ultrasound images of five histological types associated with the relevant clinical course and pathology results.

To achieve uniform image quality, which is critical as varying lesion sizes can degrade the quality of synthesized images, this study specifically targeted lesions smaller than 15 mm.

In the study, while pathology results were the gold standard for determining the histological type, some benign masses, such as typical cysts and fibroadenomas, were diagnosed as benign based on imaging findings and clinical course, without the need for pathological examination. A total of 1008 breast ultrasound images were extracted. Approximately 200 ultrasound images of breast masses were collected for each histological type. The histological types included cysts and fibroadenomas for benign masses and invasive ductal carcinoma of the breast (scirrhous, solid, and tubule-forming types) for malignant masses. These three histological types are known to comprise a large portion of invasive breast cancers [26].

Details of the data for each histological type are shown in Table 1.

The viewing software TFS-01 (Toshiba Medical Systems, Tochigi, Japan) was used to convert the ultrasound Digital Images in Communication (DICOM) images to Joint Photographic Experts Group (JPEG) figures, which were trimmed to 40 × 40 mm squares that included the chest wall in Microsoft Paint (Microsoft, Redmond, WA, USA) for analysis.

### 2.4. Image Synthesis

Image synthesis was performed on a DEEPstation DK-1000 (UEI, Tokyo, Japan) containing GeForce GTX 1080 graphics-processing unit (NVIDIA, Santa Clara, CA, USA), Core i7-8700 central processing unit (Intel, Santa Clara, CA, USA), and a Deep Analyzer graphical user interface-based deep learning tool (GHELIA, Tokyo, Japan). DCGAN was used to construct the images [23]. DCGAN represents a significant advancement in the field of generative models, particularly in image generation. In a DCGAN, the discriminator, which distinguishes between real and generated images, comprises multiple layers: strided convolution layers that reduce the spatial dimensions of the input, batch normalization layers for stabilizing learning by normalizing the input to each layer, and LeakyReLU activations which allow a small gradient when the unit is inactive, preventing the dying ReLU problem. The generator, responsible for creating images, uses convolutional-transpose layers that perform the reverse of convolution, effectively upscaling input to a larger spatial dimension. It also utilizes batch normalization layers and ReLU activations, known for their efficiency in deep networks. The unique aspect of DCGAN is the use of these strided transpose-convolution layers, enabling it to upscale a latent vector into a volume of the same shape as an image, bridging the gap between the latent space and the image space effectively [23].

**Figure 1:**
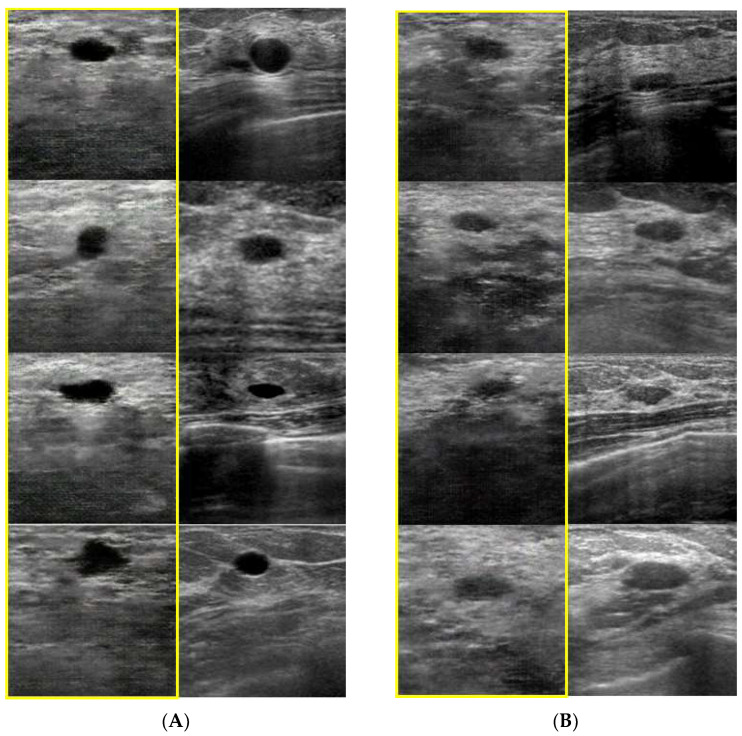

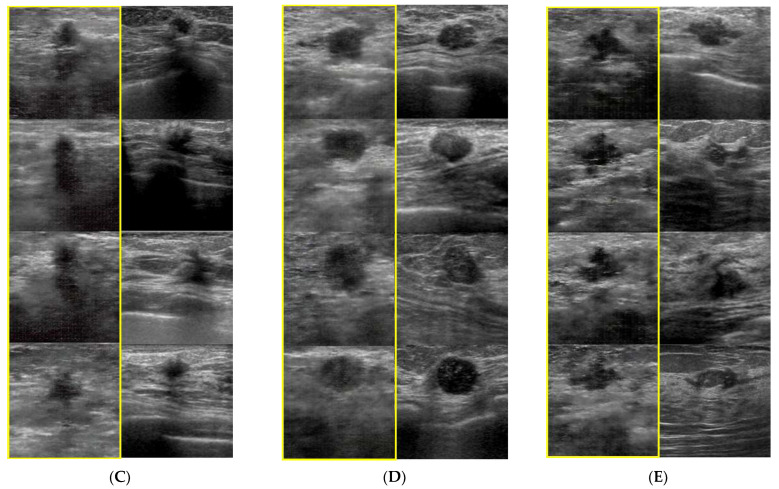
Representative generated and original images for each histological type. Figure (**A**) cyst; (**B**) fibroadenoma; (**C**) invasive ductal carcinoma (IDC)—scirrhous type; (**D**) IDC—solid type; and (**E**) IDC—tubule forming type. The images on the right are originals, and those framed in yellow on the left are synthesized (generated) images.

The parameters for the generator and discriminator were the same as those reported in previous studies [23,24,25]: optimizer algorithm = Adam (lr = 0.0002, β1 = 0.5, β2 = 0.999, eps = 8 × 10^3^). The image data were input and output at 256 × 256 pixels. After building the models, we generated 10 images with 200 epochs. In this study, a radiologist evaluated the quality of images synthesized at various epochs, and as a result, 200 epochs were selected for use. Moreover, we randomly selected 10 original images. Figure 1 shows five examples of the synthetic and original breast ultrasound images.

### 2.5. Image-Evaluation Method

Two radiologists (with 13 and 7 years of experience) were given individual reading tests using a viewer software EV Insite R (PSP Corporation, Tokyo, Japan). The readers conducted their image interpretations without knowledge of the patients’ clinical information or other imaging results, such as mammography.

The reading test consisted of a total of 50 randomly arranged synthetic images of 5 histological types (10 each): 2 benign masses (cysts and fibroadenomas) and 3 malignant masses (scirrhous, solid, and tubule-forming invasive ductal carcinoma types). The readers rated each image for (1) benign or malignant status and (2) histological type.

To minimize the impact of the first reading test, a subsequent reading test using original ultrasound images was conducted more than one month after the initial test with synthetic images. This delay was intended to reduce recall bias and ensure an unbiased evaluation of the original images.

The percentage of correct responses for the 50 questions as a whole, the percentage of benign correct responses, the percentage of malignant correct responses, and the percentage of correct responses for each histological type were calculated. Furthermore, the agreement between the two readers’ answers was assessed by determining the kappa statistic, which was calculated by comparing the two readers’ choices from five possibilities and was interpreted as follows: <0.2 slight; 0.21–0.40 fair; 0.41–0.60 moderate; 0.61–0.80 fairly high; 0.81–1.0 almost perfect.

## 3. Results

Table 1 shows the cyst group comprised 202 patients with a mean age of 50.2 years, the fibroadenoma group consisted of 201 patients with a mean age of 50.4 years, the scirrhous-type group included 201 patients with a mean age of 63.3 years, the solid-type group involved 200 patients with a mean age of 63.9 years, and the tubule-forming-type group encompassed 202 patients with a mean age of 58.4 years. Notably, the mean age of patients with malignant tumors exhibited a discernible tendency to surpass that of patients with benign tumors. Moreover, concerning mean length diameter, the three invasive ductal carcinoma types also surpassed those with benign tumors, exceeding 8.57 mm.

The percentage of correct diagnoses is shown in Table 2. For distinguishing benign or malignant cases, both readers achieved 100% accuracy with synthetic images, while their performance slightly varied with original images (94% and 96%). In identifying all histological types, reader accuracy was higher with synthetic images (86% and 78%) compared to original images (88% and 78%).

A comparison of the benign and malignant types showed that the diagnostic rates for cyst and fibroadenoma, which are benign, tended to be higher than those for malignant types. Among the malignant types, the scirrhous-type IDC tended to have a higher percentage of correct diagnoses.

The kappa coefficient, which evaluates the agreement between the two responses, was 0.650 in the reading test using the original images and 0.625 in the reading test using the synthetic images, indicating good agreement.

## 4. Discussion

In this study, the DCGAN was used to generate breast ultrasound synthetic images showing the characteristics of each pathological type. Experienced radiologists were able to identify the histological type of the synthetic images, with a positive diagnostic rate as high as that of the original ultrasound images. Furthermore, the kappa coefficient indicated good agreement between the two radiologists’ ratings based on the synthetic and original images. These results indicated that the DCGAN-generated synthetic ultrasound images had clinical properties similar to those of the original ultrasound images.

In recent years, breast imaging diagnostics have advanced significantly, with improvements in imaging technology and diagnostic accuracy [27,28,29,30,31,32]. This progress is not only due to enhancements in the quality of mammography and ultrasound images and the establishment of more refined reading techniques but also to the emergence of new modalities such as contrast-enhanced Magnetic Resonance Imaging (MRI), Positron Emission Tomography/Computed Tomography (PET/CT), and PET mammography. These advancements have made substantial contributions to the field of breast cancer detection and diagnosis. Contrast-enhanced MRI has become increasingly important in detecting breast cancer, particularly in women with dense breast tissue where traditional mammography may be less effective. This modality offers superior sensitivity in identifying malignancies, making it a valuable tool in comprehensive breast cancer screening and diagnosis. PET/CT, which combines the anatomical detail provided by Computed Tomography (CT) with the metabolic insight of Positron Emission Tomography (PET) imaging, has emerged as a powerful modality for detecting metastatic breast cancer and assessing treatment response. Similarly, PET mammography, a novel approach that integrates the high-resolution anatomical imaging of mammography with the functional imaging capabilities of PET, offers enhanced diagnostic accuracy, especially in complex cases.

These technological advancements in breast imaging have not only improved the ability to detect breast cancer at earlier stages but also enhanced the precision in characterizing tumors, thereby facilitating more personalized and effective treatment planning. The effective utilization of AI by radiologists is expected to not only improve diagnostic performance in the future but also to significantly reduce radiologists’ workload and contribute to healthcare cost savings. AI’s potential to streamline diagnostic processes and enhance accuracy promises both operational efficiency and reduced strain on healthcare systems, making it a valuable tool in modern medical practice [9,33,34,35]. GAN is one of the most remarkable methods and has been applied to medical imaging and proven useful in various areas, such as image enhancement, registration, generation, reconstruction, and transformation between images. In our previous study, the DCGAN was used to generate breast ultrasound synthetic images with a virtual complementary image of a tumor [23,24]. In this study, after adjusting the quality of the generated images, synthetic images of two benign and three malignant masses were generated, and their clinical characteristics were evaluated. We succeeded not only in generating high-quality breast ultrasound synthetic images but also in generating synthetic images based on the features of each histological type.

Breast ultrasonography is a diagnostic modality that relies on the skill and experience of the operator. Therefore, it is necessary to learn from a large number of cases to perform accurate examinations and diagnoses, and acquiring a sufficient number of cases requires much time and money, which is problematic. In addition, to improve the diagnostic accuracy for rare diseases, it is necessary to share these cases among multiple medical institutions to maintain learning opportunities and training in diagnostic images of diseases rarely encountered. However, there are limitations in data handling, as the use of test results and medical data for medical research requires the patient’s explicit consent from the standpoint of privacy protection [36]. The ethical issue of protecting patient privacy often restricts the sharing of real patient images for purposes of medical research.

Previous studies have used medical synthetic images created by GANs to train convolutional neural networks [37,38,39], so the use of GANs may reduce the cost and time of data collection and reduction and solve the problem of insufficient datasets in case learning. Furthermore, based on this study’s finding that breast ultrasound images generated by GAN have the same clinical characteristics as the original examination images, the learning effect of training with secondary images by GAN may be as effective as the learning effect from examining original images. In summary, using GAN-generated images for medical learning overcomes privacy issues and can greatly facilitate the sharing of research data to enhance research and the training of physicians, which will help maintain the expertise needed for highly accurate diagnosis, including in rare cases, and improve knowledge in the use of these research and training techniques. This methodology can enhance trainees’ exposure to a diverse range of cases, including rare pathologies, thereby broadening their diagnostic expertise and adaptability in clinical settings. It is probable that the use of GAN-synthesized breast ultrasound images will increase at an ever-accelerating pace.

There were several limitations in this study that should be considered. First, this retrospective study was conducted at a single institution. The fidelity of image quality may exhibit variability attributed to the age of ultrasound equipment and disparities among ultrasound examination manufacturers. While this study excluded images with artifacts from the collected dataset, it remains conceivable that their presence could exert an influence on the quality of the generated images. Therefore, a larger multicenter study is needed to assess the validity of our study. Similarly, although the original images collected in this study were trained on a relatively small number of cases, we believe that it will be possible to use a larger volume of clear noise-free images of the lesion area with the ultrasound image artifacts removed to create more sophisticated generated images. Second, not all of the patients whose original images were used in this study were pathologically diagnosed. Therefore, the final histopathological findings may differ from the diagnosis given by the physician at the time of the ultrasound examination, which was used to select the patients in this study. Third, this study was limited to five representative masses observed in breast tissue. Therefore, exploring other histological types of breast masses in future research could potentially expand the clinical application of synthetic images generated by GANs in a broader range of scenarios. Subsequent research endeavors will conduct supplementary experiments to enhance the precision performance in image synthesis, employing well-balanced datasets.

## 5. Conclusions

The synthetic images generated by the DCGAN accurately represented the characteristics of each of the five histological types studied, demonstrating that the physician readers could make diagnoses similar to those made from the original images. The use of GAN-synthesized breast ultrasound images in medical education offers an innovative approach to training, circumventing limitations imposed by privacy concerns and data scarcity.

## Figures and Tables

**Table 1:** Ultrasound image data by histological type.

Histologic Type	Number of Images [n]	Long Diameter of Masses		Patient Age	
		Range [mm]	Average [mm]	Range [y]	Average [y]
Cyst	202	4–11	6.84	28–84	50.2
Fibroadenoma	203	5–12	7.79	20–85	50.4
IDC—Scirrhous type	201	5–12	8.57	40–89	63.3
IDC—Solid type	200	5–14	8.87	33–90	63.9
IDC—Tubule-forming type	202	5–13	9.41	33–89	58.4

IDC; Invasive ductal carcinoma.

**Table 2:** Percentage of correct diagnoses by readers (reading tests using either synthetic or original images (each set interpreted 1 month apart).

Correct Diagnosis Rate	Synthetic Image		Original Image	
	Reader 1	Reader 2	Reader 1	Reader 2
Benign or malignant	50/50 100%	47/50 94.0%	50/50 100%	48/50 96.0%
All histological types	43/50 86.0%	39/50 78.0%	44/50 88.0%	39/50 78.0%
Cyst	9/10 90.0%	9/10 90.0%	9/10 90.0%	7/10 70.0%
Fibroadenoma	9/10 90.0%	10/10 100%	9/10 90.0%	10/10 100%
IDC—Scirrhous type	10/10 100%	6/10 60.0%	9/10 90.0%	9/10 90.0%
IDC—Solid type	9/10 90.0%	7/10 70.0%	9/10 90.0%	6/10 60.0%
IDC—Tubule forming type	6/10 60.0%	7/10 70.0%	8/10 80.0%	7/10 70.0%

IDC; Invasive ductal carcinoma.

## Data Availability

Data are contained within the article.
